# Facing the digital divide into a dementia clinic during COVID-19 pandemic: caregiver age matters

**DOI:** 10.1007/s10072-020-05009-w

**Published:** 2021-01-18

**Authors:** Andrea Arighi, Giorgio Giulio Fumagalli, Tiziana Carandini, Anna Margherita Pietroboni, Milena Alessandra De Riz, Daniela Galimberti, Elio Scarpini

**Affiliations:** 1 Neurodegenerative Diseases Unit, Fondazione IRCCS Ca’ Granda Ospedale Maggiore Policlinico, Via Francesco Sforza 35, 20122 Milan, Italy; 2 University of Milan, “Dino Ferrari” Center, Milan, Italy

**Keywords:** Dementia, Caregiver, Telemedicine, Digital divide, Digital native

## Abstract

**Background:**

The coronavirus disease 2019 (COVID-19) pandemic has dramatically stressed the health care system and has provoked changes in population use of digital technologies. Digital divide is any uneven distribution in Information and Communications Technologies between people.

**Aims:**

The purpose of this work was to describe the digital divide of a population of patients with dementia contacted by telemedicine during Italian lockdown for COVID-19 pandemic.

**Method:**

One hundred eight patients with cognitive impairment were contacted by video call to perform a telemedicine neurological evaluation. Information on patients and caregivers attending the televisit were recorded.

**Results:**

Seventy-four patients connected with neurologist (successful televisit, 68.5%) and 34 patients were not able to perform televisit and were contacted by phone (failed televisit, 31.5%). No significant differences were observed among the two groups concerning age, gender, and education, but the prevalence of successful televisit was higher in the presence of younger caregivers: televisits performed in the presence of subjects of younger generation (sons and grandsons) had a successful rate higher (86% successful, 14% failed) than the group without younger generation caregiver (49% successful, 51% failed). This difference is mainly due to the ability of technological use among younger people.

**Discussion:**

The most impacting factors on digital divide in our population are the social support networks and the experience with the technology: the presence of a digital native caregiver. The COVID-19 pandemic is unmasking an emerging form of technology-related social inequalities: political and community interventions are needed to support the most socially vulnerable population and prevent social health inequalities.

## Introduction

Over the past few months, the coronavirus disease 2019 (COVID-19) pandemic has dramatically stressed the health care system. The rapid rate of COVID-19-related patient illnesses has caused the Ministry of Health to recommend postponing or canceling outpatient visits of patients with chronic diseases. Therefore, clinicians had to find strategies to deal with the management of their patients’ problems during the lockdown: the rate at which medical practices have had to transition to telemedicine visits is just as fast as the rate at which COVID-19 is surging through the world [1]. From a technological perspective, the COVID-19 pandemic has provoked massive, immediate, and unprecedented changes in population use of digital technologies and media [2]. Online technologies became the privileged channel for governments and supranational entities such as the World Health Organization to convey their messages and recommendations. More importantly, technology is becoming central to maintain active social interactions [3]. This rapid transition has made it difficult for physicians and patients to anticipate barriers to successfully implement telemedicine visits, facing with digital divide.

Digital divide is any uneven distribution in the access to, use of, or impact of Information and Communications Technologies (ICT) between any number of distinct groups, which can be defined based on social, geographical, or geopolitical criteria, or otherwise [4]. Access to the Internet and the spread of broadband are some of the prerequisites for the spread of ICT among the population. The COVID-19 pandemic crisis exacerbates the importance of a hidden form of social inequality and digital inequalities [3]. Four factors are impacting to the degree of ability to use technologies efficiently and effectively [5]: (1) the quality of the equipment, (2) autonomy of use, (3) social support networks, and (4) experience with the technology for retaining benefits from its use.

In 2019, in Italy, 76.1% of families had access to Internet, and 74.7% had a broadband connection. Strong digital divide is evident between families, mainly due to generational and cultural factors: almost all families with at least one minor have a broadband connection (95.1%); among families made up exclusively of people over 65, this share falls to 34.0% [6].

Due to the alarming COVID-19 spreading, from the second week of March, Italian Ministry of Health has stopped outpatient visits for chronic patients, unless for emergency. As a consequence, in the following months, neurologists have implemented telemedicine services to deal with the management of patients with dementia. The purpose of this work was to describe the digital divide of a population of patients with dementia contacted by telemedicine during the lockdown due to the COVID-19 pandemic and to understand which factors can influence telemedicine successfulness (i.e., demographic and cognitive data of the subjects, presence, and generation of caregivers).

## Materials and methods

Subjects were enrolled in the study consecutively from mid-April (when hospital implemented telemedicine) to the end of July 2020 at the Alzheimer Centre of the Fondazione IRCCS Ca’ Granda Ospedale Maggiore Policlinico of Milan (Italy).

We proposed telemedicine visit using Microsoft® Teams (https://www.microsoft.com) to patients with dementia, afferent to our outpatient service, who had to carry out the follow-up visit during the lockdown time, between March 9 and the end of July. Informed consent approved by the local Institutional Review Board was obtained from all included patients, in accordance with specific national laws, and instructions to connect to Microsoft® Teams were sent by mail, after phone contact by administrative staff to propose telemedicine. The software enabled neurologists to communicate with patients from their workstations, starting a video call, and using a headset. During the televisit, neurologists collected patients’ sociodemographic information and the characteristics of the caregiver who assisted the patient. After that, the neurologist collected recent medical history, performed a brief neurological examination according to telehealth and remote care advices from the American Academy of Neurology (https://www.aan.com/tools-and-resources/practicing-neurologists-administrators/telemedicine-and-remote-care/), and either provided advices and prescriptions or modified the therapy. The patient’s caregiver was contacted by phone for a quick assessment in case the connection could not be established through the software. Neurologists recorded whether the connection during televisit was successful or failed, considering only presence or absence of a proper connection to communicate.

The data obtained from the study were analyzed using statistical analysis software Jamovi (https://www.jamovi.org/). Frequency and percentage were used for categorical data, and mean and standard deviation values were used for continuous data. Chi-square test was used in the analysis of categorical data, and independent sample *t* test was used in the analysis of continuous data. The tests were examined at a 95% confidence level, and significant differences were interpreted as a result of the tests. Values with *p* < 0.05 were considered to be statistically significant.

## Results

Data from 108 patients with cognitive impairment were considered. Subjects enrolled in the study were categorized into two groups based on the successfulness of televisits: 74 patients successfully connected with neurologist on Microsoft Teams (successful televisit, 68.5%), and 34 patients failed to perform televisit and were subsequently contacted by phone (failed televisit, 31.5%). The reasons for connection failure were the lack of PC, tablet, or phone with Internet connection in 8 cases (23.5%) and the difficulty in establishing a connection in 26 cases (76.4%).

Sociodemographic and clinical characteristics of patients are summarized in Table 1. No significant differences were observed among the two groups concerning age, gender, education, and Mini Mental State Examination (MMSE) from previous in person visit (Table 1).

**Table 1:** Demographic and clinical characteristics of patients, divided according to televisit outcome, and comparison between groups

	Successful televisit	Failed televisit	*p*
Age (years) mean (SD)	73.5 (7.15)	75.7 (7.2)	0.135
Gender M:F (% of M)	38:36 (51.4)	14:20 (41.2)	0.326
Education (years) mean (SD)	11.1 (4.39)	11.8 (4.66)	0.441
MMSE mean (SD)	18.7 (8.47)	19.8 (9.32)	0.552

*SD* Standard Deviation, *MMSE* Mini Mental State Examination Independent sample *t* test was used in the analysis of continuous data (age, education, MMSE); chi-square test was used in the analysis of categorical data (gender)

**Fig. 1:**
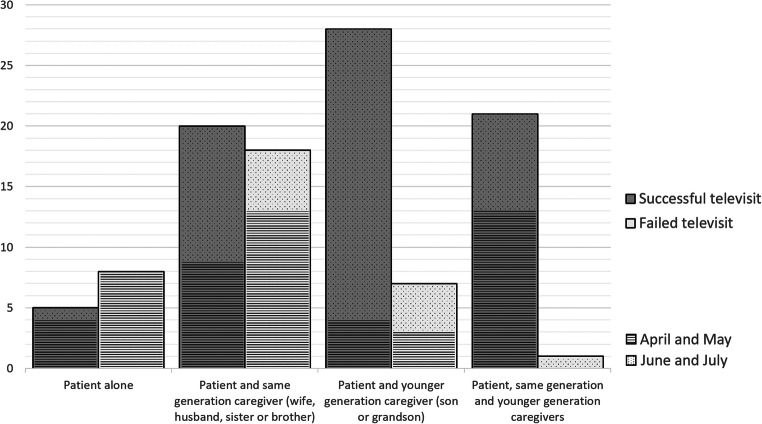
Number of performed televisits distributed according to presence of caregiver and to outcome (bars are divided according to timing: lines for televisits performed in April and May, dots for televisits performed in June and July)

Stratifying subjects, according to characteristics of the caregiver who assists the patient during the televisit, four groups have been obtained: (1) patient alone; (2) patient and caregiver of the same generation (wife, husband, sister, or brother); (3) patient and caregiver of the following generation (son or grandson); and (4) patient, caregiver of the same generation, and caregiver of the younger generation. Data are summarized in Table 2 and Fig. 1. We could not further stratify participants in sons and grandsons because of the low number of the grandson group (50 televisits with sons, 5 with grandsons, and 2 with sons and grandsons together).

**Table 2:** Modality of televisits (presence of caregiver and timing), divided according to outcome

Period	Group	Total	Successful televisit	Failed televisit	Significance
From April to July	Patient alone	13	5 (38.5%)	8 (61.5%)	a, b*
	Patient and same generation caregiver (wife, husband, sister, or brother)	38	20 (52.6%)	18 (47.4%)	a, b*
	Patient and younger generation caregiver (son or grandson)	35	28 (80%)	7 (20%)	c, d
	Patient, same generation caregiver, and younger generation caregiver	22	21 (95%)	1 (4.5%)	c*, d*
April and May	Patient alone	12	4 (33%)	8 (66.7%)	b*
	Patient and same generation caregiver (wife, husband, sister, or brother)	22	9 (40.9%)	13 (59.1%)	b*
	Patient and younger generation caregiver (son or grandson)	7	4 (57.1%)	3 (42.9%)	b
	Patient, same generation caregiver, and younger generation caregiver	13	13 (100%)	0 (0%)	c*, d*, a
June and July	Patient alone	1	1 (100%)	0 (0%)	–
	Patient and same generation caregiver (wife, husband, sister, or brother)	16	11 (68.8%)	5 (31.3%)	–
	Patient and younger generation caregiver (son or grandson)	28	24 (85.7%)	4 (14.3%)	–
	Patient, same generation caregiver ,and younger generation caregiver	9	8 (88.9%)	1 (11.1%)	–

Data are reported as absolute number of televisits (percentage). The *χ*2 was used for comparison of each group frequency according to the legend:

a: vs Patient and younger generation caregiver (son or grandson)

b: vs Patient, same generation caregiver, and younger generation caregiver

c: vs Patient alone

d: vs Patient and same generation caregiver (wife, husband, sister, or brother)

*p < 0.001; otherwise, *p* < 0.05

Among groups the prevalence of successful televisit was higher in presence of caregiver of younger generation: 80% for patient with caregiver of the younger generation and 95% for patient with caregiver of the same generation and younger generation (Table 2, Fig. 1). Chi-square tests between groups are summarized in Table 2.

**Fig. 2:**
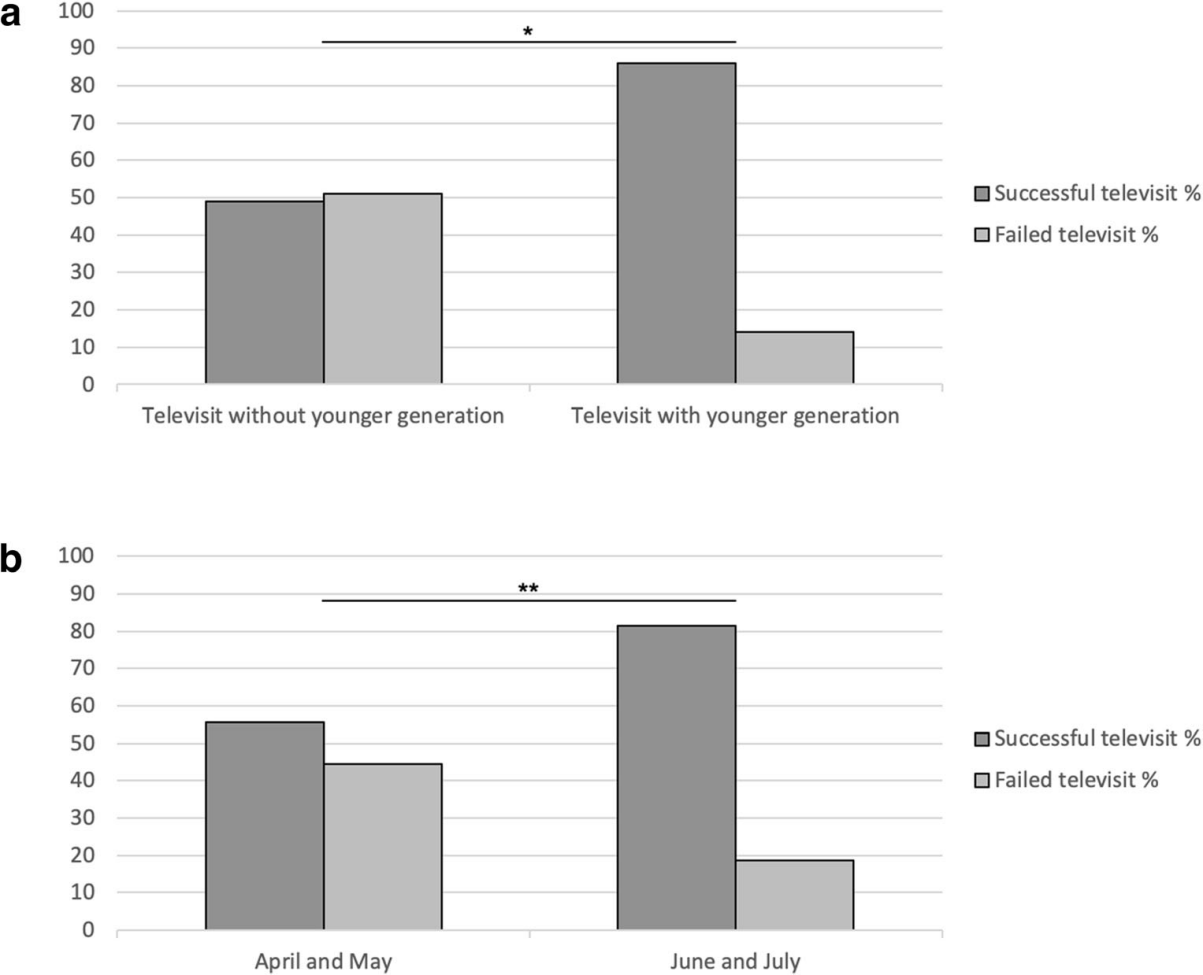
(**a**) Successful rate confrontation between televisits performed without and with younger generation caregiver. (**b**) Successful rate confrontation between televisits performed in April/May and June/July. * *p* < 0.001, ** *p* = 0.004

Stratifying patients according to the presence of a caregiver of the younger generation, two groups have been obtained: 51 patients underwent televisit without caregiver of younger generation (49% successful televisit, 51% failed televisit), and 57 patients underwent televisit with caregiver of younger generation (86% successful televisit, 14% failed televisit) (Fig. 2a). Televisits performed in the presence of subjects of younger generation had a successful rate higher than the group without younger generation caregiver. Chi-square test between these two groups resulted statistically significant (*p* < 0.001).

According to data of execution of televisit, subjects were divided in two equal group: 54 patients underwent televisit during April and May and 54 patients during June and July. Chi-square test showed that televisit successful rate was different between these two groups (April and May 55.6%, June and July 81.5%, *p* = 0.004) (Fig. 2b).

Variables resulting significantly related to televisit success (the presence of a caregiver of the younger generation and the period of televisit execution) were then included as binary independent variables in a logistic regression model exploring their association with televisit outcome (successful televisit vs. failed televisit, binary dependent variable). Period of televisit execution did not result significantly associated with televisit success (*p* = 0.069), while the presence of a caregiver of the younger generation significantly influenced televisit success (*p* < 0.001, OR 5.14, confidence interval 1.98–13.38).

## Discussion

In this paper, we described digital divide in a population of patients with dementia of an outpatient service in Milan, a big Italian city with one of the highest prevalence of cases during the COVID-19 pandemic. 31.5% of the patients failed to perform telemedicine, and this was not due to the lack of tools (only 23.5% of these subjects declared the absence of available hardware with Internet connection) but instead for connection difficulties. These data are consistent with data from ISTAT (Italian National Institute of Statistics) concerning Italian general population showing that 76.1% of families had access to Internet, but they are far above the percentage described in people over 65 (only 34.0% have access to Internet). This discrepancy is mainly due to population characteristics: our patients mainly reside in a large city of northern Italy, where even the elderly population is likely to have easier access to Internet.

Successfulness in televisit was not related to sociodemographic and clinical characteristics of patients but was significantly associated to features of caregivers: patients who were supported by a caregiver of the younger generation (son or grandson) had a telemedicine higher successful rate. This evidence is mainly due to the ability of technological use among younger people; in fact sons and grandsons of our patients cohort are almost all digital natives (a person who has grown up in the digital age), while caregivers of the same generation of patients are all digital immigrants (people who has acquired familiarity with digital systems as an adult) [7].

The successfulness in televisit was also found to be related to the months in which televisit was performed, possibly due to the fact that during the lockdown, general digital capabilities have been enhanced, to satisfy the demand of different online activities (shopping, meeting, etc.). From a technological perspective, the COVID-19 pandemic has provoked massive, immediate, and unprecedented changes in population use of digital technologies and media [2]. The simplest explanation is that at the end of the lockdown, sons and grandsons were able to reach the patients’ home more easily. As can be seen in Fig. 1, stratifying televisits according to the period of execution, it is observed that in the second part of the lockdown, fewer patients performed the visit alone, while the presence of a young family member with the patient was more frequent, due to minor restrictions of lockdown rules. This theory is supported by regression analyses: the period of televisit execution was not significantly associated with televisit success, while the presence of a caregiver of the younger generation significantly influenced televisit success.

According to the Hargittai model [5], the factors most impacting on digital divide in our population are the social support networks and the experience with the technology: the presence of a digital native caregiver. The COVID-19 pandemic is unmasking an emerging form of technology-related social inequalities that were rampant since some times already, but did not receive the full attention it deserved. This crisis represents the first large-scale event for which digital inequalities become a major factor of vulnerability. A series of political and community interventions aiming to solidify the social safety net is needed to support the most socially vulnerable population and prevent increasing both their vulnerability to the pandemic and the social health inequalities [3].

More importantly than providing patients with a PC, tablet, or phone with Internet connection, this study highlights the urgent need of educating them on the use of electronic devices. Patient and caregivers lack of knowledge, unfamiliarity with communication technology, and fear of the unknown are well-known causes for lack of adoption to telemedicine [8, 9]. Education programs and training sessions can ease patients’ anxiety and enhance their experience with telemedicine visits [1]. Italian Parliament and the European Council identify digital skills as one of the eight key competences for lifelong learning, aimed at acquiring knowledge that lasts over time and necessary for every citizen to be able to fit into the social and work environment [10].

The major limitations of the present study are the small number of participants and the lack of accurate sociodemographic and socioeconomic information of caregivers. A multicenter study with larger groups of patients and caregivers is needed to confirm these preliminary data. Another limitation is that we did not check caregivers’ satisfaction with questionnaires following televisits.

## Conclusion

Digital divide for telemedicine characterized almost 30% of patients with dementia from a large clinic in a city in Northern Italy. The age of the caregiver was found to influence the successfulness of the televisit, and this factor should always be considered when arranging a telemedicine appointment. Political and community interventions are needed to support the most socially vulnerable population and prevent social health inequalities.
